# The power of tumor sizes in predicting the survival of solitary hepatocellular carcinoma patients

**DOI:** 10.1002/cam4.1873

**Published:** 2018-11-14

**Authors:** Anli Yang, Weikai Xiao, Dong Chen, Xiaoli Wei, Shanzhou Huang, Ye Lin, Chuanzhao Zhang, Jianwei Lin, Feiwen Deng, Chenglin Wu, Xiaoshun He

**Affiliations:** ^1^ Department of Breast Oncology, Sun Yat‐sen University Cancer Center, State Key Laboratory of Oncology in South China Collaborative Innovation Center for Cancer Medicine Guangzhou China; ^2^ Organ Transplant Center The First Affiliated Hospital of Sun Yat‐sen University Guangzhou China; ^3^ Department of Pancreatobiliary Surgery The First Affiliated Hospital of Sun Yat‐sen University Guangzhou China; ^4^ Department of Medical Oncology, Sun Yat‐sen University Cancer Center, State Key Laboratory of Oncology in South China Collaborative Innovation Center for Cancer Medicine Guangzhou China; ^5^ Department of General Surgery, Guangdong General Hospital Guangdong Academy of Medical Sciences Guangzhou China

**Keywords:** liver resection, liver transplantation, prognosis, radiofrequency ablation, solitary hepatocellular carcinoma, tumor sizes

## Abstract

**Background:**

Vascular invasion, rather than tumor size, was applied into the 7th edition of the AJCC TNM staging system to predict survival of solitary hepatocellular carcinoma (HCC) patients. However, does this mean tumor size is of little value in prognostic prediction? The current study was designed to explore the prognostic ability of tumor sizes in solitary HCC.

**Methods:**

A total of 18 591 patients with solitary HCC categorized as T1 and T2 were retrieved from the Surveillance Epidemiology and End Results (SEER) database. The Cox proportional hazards regression model was adopted to evaluate the impact of tumor sizes on overall survival (OS) and disease‐specific survival (DSS) in general and in subgroups stratified by vascular invasion and surgery type.

**Results:**

Large tumor sizes (>39 mm) were associated with unfavorable clinicopathologic characteristics. Compared with tumors ≤30 mm, tumors between 31‐50 mm and tumors >50 mm showed significantly worse OS and DSS in general using multivariate analysis (all *P* < 0.001). In subgroup analyses, for patients without vascular invasion, tumor size was a notable prognostic indicator for OS in the radiofrequency ablation group (*P* < 0.001), rather than in the liver resection or transplantation group. Nevertheless, for patients with vascular invasion, tumor sizes exhibited a notable impact on OS in the liver resection and transplantation group.

**Conclusions:**

The AJCC TNM staging system for solitary HCC would be more comprehensive if tumor sizes were integrated into the T2 classification. Additionally, for T1 patients, tumor sizes play no role in the choice between resection and transplantation.

## 1 INTRODUCTION

As the second largest contributor to cancer‐related mortality, hepatocellular carcinoma (HCC) ranked sixth among the most commonly occurring cancers in the world.^1^ In the United States, the age‐adjusted incidence of HCC was at least 6/100 000 in 2010,^2^ and its morbidity has continued rising recently.^3^ With increased surveillance of high‐risk populations, the proportion of early‐staged HCC has increased dramatically in recent years.^4^, ^5^ Radiofrequency ablation (RFA), liver resection (LR), and liver transplantation (LT) are three major treatments for HCC.^3^ The selection of therapy had profound effects on HCC prognosis, which therefore should be made after comprehensive and careful consideration.

There are several staging systems applied in HCC, among which the Barcelona Clinic Liver Cancer (BCLC) classification and American Joint Committee on Cancer (AJCC) tumor‐node‐metastasis (TNM) staging systems are most widely recognized. Numerous factors have been identified as prognostic indicators and are incorporated into various staging systems. These factors include lesion numbers,^6^ vascular invasion,^7^, ^8^, ^9^ portal hypertension,^10^, ^11^ regional lymph node metastases,^12^, ^13^, ^14^ distant metastases, laboratory examination indicators such as serum albumin^15^, ^16^, ^17^ and bilirubin,^15^, ^16^ and so on. Tumor size has been included in several staging systems, such as the Liver Cancer Study Group of Japan TNM staging system^18^ and the Hong Kong staging system.^19^ However, it has been partially removed from the 6th and 7th editions of the AJCC TNM staging system because of its controversial prognostic ability in HCC.^20^

In the 7th edition of the AJCC TNM staging system, 50 mm was the cut‐off point in dividing multiple lesions HCC into T2 (none larger than 50 mm) and T3 (at least one >50 mm) classifications. However, in terms of solitary HCC, tumor size was not included in T classification. Instead, vascular invasion was used to divide HCC with solitary tumor into T1 (no vascular invasion) and T2 (with vascular invasion) classifications. With data retrieved from the Surveillance Epidemiology and End Results (SEER) database, this study aims to explore the prognostic ability of tumor sizes for HCC with solitary tumor stratified by vascular invasion and surgery type.

## 2 MATERIALS AND METHODS

### 2.1 Ethics statement

This study was deemed exempt from institutional review board approval by The First Affiliated Hospital of Sun Yat‐sen University, thus informed consent was waived. The study was conducted in accordance with the ethical standards of the World Medical Association's Declaration of Helsinki.

### 2.2 Database and patient selection

The SEER database (https://seer.cancer.gov/data/) consists of 18 population‐based cancer registries in the United States and it represents approximately 16% of the whole population. As the largest publicly available cancer dataset worldwide, SEER research data contains demographic information and several clinicopathologic characteristics.

**Figure 1:**
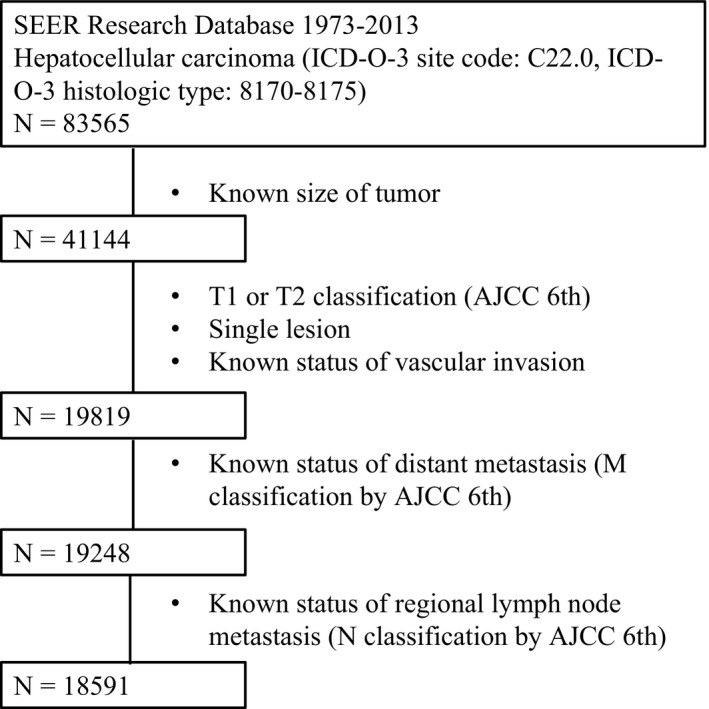
The flow chart for selection of study population. SEER, Surveillance, Epidemiology, and End Results; ICD‐O‐3, international classification of diseases for oncology, 3rd edition; AJCC, American Joint Committee on Cancer

Pathologically diagnosed HCC (ICD‐O‐3 site code: C22.0, ICD‐O‐3 histologic type: 8170‐8175) from 1973 to 2013 was extracted from the SEER database. There were 83,565 cases in total. Among them, only those with known tumor sizes were included (N = 41 144). After that, patients with single lesions, known status of vascular invasion, and T1 or T2 classifications (AJCC 6th) were further selected into the study population (N = 19 819). Finally, subjects with missing data for M classification (AJCC 6th, N = 517) and N classification (AJCC 6th, N = 657) were excluded, leaving 18,591 HCC patients enrolled as the final study population. Detailed inclusion and exclusion criteria are described in Figure 1.

### 2.3 Statistical analyses

Statistical analyses were all performed with SPSS for Windows V.13.0. (SPSS Inc, Chicago, IL, USA). Comparisons of characteristics between groups were conducted by chi‐square test or Kruskal–Wallis H test. The latter was used for ordinal variables. Overall survival (OS) was defined as the time interval between diagnosis of HCC and death attributed to any cause, while disease‐specific survival (DSS) was defined as the time interval between diagnosis of HCC and death attributed to HCC only. Univariate and multivariate Cox proportional hazards regression models were applied to evaluate predictive values of tumor sizes for OS and DSS.^21^ The hazard ratio (HR) or adjusted hazard ratio (AHR) and 95% confidence interval (CI) were estimated by the Cox proportional hazards model. Adjusted survival curves were plotted by the Kaplan‐Meier method and compared by log‐rank test. *P* values ≤0.05 were considered to indicate statistical significance.

## 3 RESULTS

### 3.1 Demographic and clinicopathologic association of tumor sizes

A total of 18,591 patients with solitary HCC classified as T1 or T2 by the AJCC 6th staging system were included in the final study population, of which the median tumor size was 39 mm. The study population was then divided into two subgroups: small tumor sizes (≤39 mm) and large tumor sizes (>39 mm). Demographic and clinicopathologic characteristics were compared between the two aforementioned subgroups of patients (Table 1). Large tumor sizes (>39 mm) were found to be associated with male gender (*P* < 0.001), older age (*P* < 0.001), lower fibrosis score (*P* < 0.001), and lower rates of RFA and LT treatment (*P* < 0.001). Moreover, large tumor sizes (>39 mm) were associated with many unfavorable characteristics, including regional lymph node metastases (*P* < 0.001), distant metastases (*P* < 0.001), vascular invasion (*P* < 0.001), elevated alpha‐fetoprotein (AFP) level (*P* < 0.001), and higher rate of widowed marriage status (*P* < 0.001). Detailed information is shown in Table 1.

**Table 1:** Demographic and clinicopathologic relevance of tumor size in solitary hepatocellular carcinoma

Characteristics	Tumor size (median: 39 mm)		*P* value
	≤39	>39	
Gender			
Male	6695 (71.9)	6946 (74.8)	<0.001
Female	2614 (28.1)	2336 (25.2)	
Age (median: 63 y)			
≤63	5727 (61.5)	4145 (44.7)	<0.001
>63	3582 (38.5)	5137 (55.3)	
Year of diagnosis			
2004‐2007	2391 (25.7)	3030 (32.6)	<0.001
2008‐2010	2945 (31.6)	2846 (30.7)	
2011‐2013	3973 (42.7)	3406 (36.7)	
N classification			
N0	9150 (98.3)	8822 (95.0)	<0.001
N1	159 (1.7)	460 (5.0)	
M classification			
M0	9059 (97.3)	8339 (89.8)	<0.001
M1	250 (2.7)	943 (10.2)	
Vascular invasion			
No	8476 (91.1)	8072 (87.0)	<0.001
Yes	833 (8.9)	1210 (13.0)	
AFP level			
Normal and Borderline	2633 (35.5)	2149 (30.5)	<0.001
Elevated	4780 (64.5)	4900 (69.5)	
Unknown (n = 4129)			
Fibrosis score			
0‐4	568 (17.1)	671 (32.8)	<0.001
5‐6	2749 (82.9)	1373 (67.2)	
Unknown (n = 13230)			
Surgery type			
No surgery	4612 (49.7)	6318 (68.4)	<0.001
RFA	1850 (19.9)	527 (5.7)	
Liver resection	1152 (12.4)	1822 (19.7)	
Liver transplantation	1252 (13.5)	253 (2.7)	
Other surgery	416 (4.5)	317 (3.4)	
Unknown (n = 72)			
Race			
American Indian/Alaska Native	102 (1.1)	99 (1.1)	0.01
White	6457 (69.4)	6275 (67.6)	
Black	1103 (11.8)	1125 (12.1)	
Asian or Pacific Islander	48 (0.5)	1761 (19.0)	
Unknown (n = 70)			
Marriage status			
Widowed	786 (8.9)	1145 (12.8)	<0.001
Divorced	1219 (13.7)	963 (10.8)	
Separated	199 (2.2)	132 (1.5)	
Single	1805 (20.3)	1708 (19.2)	
Married	4872 (54.9)	4964 (55.7)	
Unknown (n = 798)			
Insurance status			
Uninsured	207 (3.3)	256 (4.4)	0.001
Insured	6130 (96.7)	5498 (95.6)	
Unknown (n = 6500)			

AFP, alpha‐fetoprotein; RFA, radiofrequency ablation.

### 3.2 Prognostic ability of tumor sizes in general population

The general population was categorized into three subgroups with two cut‐off points in tumor sizes, 30 mm and 50 mm, which were adopted to define small HCC and acted as tumor size limitations between RFA and LT.^22^, ^23^, ^24^ Consequently, there were three subgroups of patients: ≤30 mm group, 31‐50 mm group, and >50 mm group. The median OS of the ≤30 mm group was 46.0 months with 95% CI of 43.4‐48.6 months, the median OS of the 31‐50 mm group was 26.0 months with 95% CI of 24.5‐27.5 months, and the median OS of the >50 mm group was 10.0 months with 95% CI of 9.4‐10.6 months. In univariate analysis, compared with the ≤30 mm group, the other two groups had inferior OS, with HR and 95% CI as 1.48 (1.41‐1.56) and 2.48 (2.37‐2.60), respectively. Other remarkable prognostic factors identified by univariate analyses included age (≤63/>63 years), year of diagnosis (2004‐2007/2008‐2010/2011‐2013), N classification (N0/N1), M classification (M0/M1), AFP level (Normal and Borderline/Elevated), fibrosis score (0‐4/5‐6), surgery type (No surgery/RFA/LR/LT/Other surgery), race ([American Indian/Alaska Native]/White/Black/Asian or Pacific Islander), marriage status (Widowed/Divorced or separated or single/Married), and insurance status (Uninsured/Insured) (almost all *P* < 0.001).

**Figure 2:**
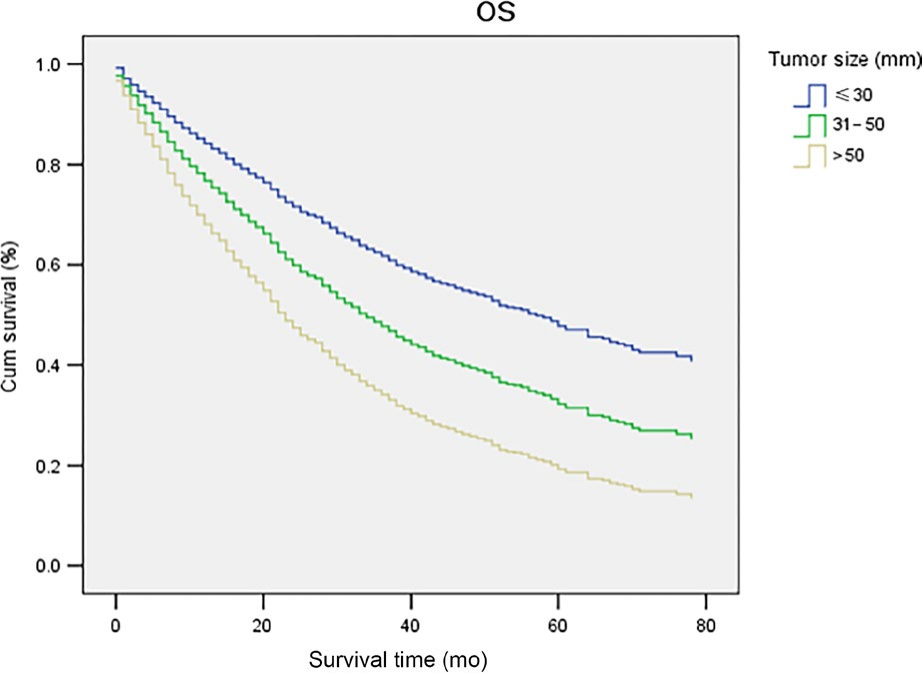
Adjusted OS of patients with solitary HCC by tumor size (≤30 mm/31‐50 mm/>50 mm) in the whole study population. Compared with tumors ≤30 mm, tumors between 31‐50 mm had inferior adjusted OS in multivariate analyses (AHR and 95% CI: 1.53 [1.35‐1.75]), and tumors >50 mm also showed worse adjusted OS in multivariate analyses (AHR and 95% CI: 2.23 [1.94‐2.56]). HCC, hepatocellular carcinoma; OS, overall survival; AHR, adjusted hazard ratio; CI, confidence interval

After being adjusted by the above prognostic factors in multivariate analyses, it was found that tumor size was an independent and pronounced prognostic factor for OS. Compared with the ≤30 mm group, the other two groups had inferior OS in multivariate analyses, with AHR and 95% CI as 1.53 (1.35‐1.75) and 2.23 (1.94‐2.56), respectively. The adjusted survival curves for OS stratified by tumor sizes are shown in Figure 2. Other independent and marked prognostic factors for OS included year of diagnosis (2004‐2007/2008‐2010/2011‐2013), N classification (N0/N1), M classification (M0/M1), AFP level (Normal and Borderline/Elevated), fibrosis score (0‐4/5‐6), surgery type (No surgery/RFA/LR/LT/Other surgery), and race ([American Indian/Alaska Native]/White/Black/Asian or Pacific Islander). Details of univariate and multivariate analyses on OS in the general population are shown in Table 2.

**Table 2:** Univariate and multivariate Cox proportional hazards regression analyses for the impact of tumor size on OS

Characteristics	Number (%)	Univariate		Multivariate	
		HR (95% CI)	P value	Adjusted HR (95% CI)	*P* value
Gender (Male/Female)	13641 (73.4)/4950 (26.6)	0.98 (0.94‐1.02)	0.35		
Age (≤63/>63)	9872 (53.1)/8719 (46.9)	1.48 (1.42‐1.54)	<0.001	1.28 (1.15‐1.43)	<0.001
Year of diagnosis					
2004‐2007	5421 (29.2)	1 (reference)	<0.001	1 (reference)	0.02
2008‐2010	5791 (31.1)	0.95 (0.91‐1.00)	0.04	0.81 (0.68‐0.95)	0.01
2011‐2013	7379 (39.7)	0.85 (0.81‐0.90)	<0.001	0.77 (0.64‐0.92)	0.004
Tumor size (mm)					
≤30	7119 (38.3)	1 (reference)	<0.001	1 (reference)	<0.001
31‐50	5006 (26.9)	1.48 (1.41‐1.56)	<0.001	1.53 (1.35‐1.75)	<0.001
>50	6466 (34.8)	2.48 (2.37‐2.60)	<0.001	2.23 (1.94‐2.56)	<0.001
N classification (N0/N1)	17972 (96.7)/619 (3.3)	2.39 (2.18‐2.61)	<0.001	1.40 (1.03‐1.90)	0.03
M classification (M0/M1)	17398 (93.6)/1193 (6.4)	3.55 (3.32‐3.78)	<0.001	2.78 (2.22‐3.49)	<0.001
Vascular invasion (No/Yes)	16548 (89.0) /2043 (11.0)	0.96 (0.90‐1.02)	0.16		
AFP level (Normal and Borderline/Elevated)	4782 (33.1)/9680 (66.9)	1.55 (1.48‐1.63)	<0.001	1.40 (1.25‐1.58)	<0.001
Fibrosis score (0‐4/5‐6)	1239 (23.1)/4122 (76.9)	1.28 (1.17‐1.41)	<0.001	1.35 (1.16‐1.58)	<0.001
Surgery type					
No surgery	10930 (59.0)	1 (reference)	<0.001	1 (reference)	<0.001
RFA	2377 (12.8)	0.41 (0.39‐0.44)	<0.001	0.54 (0.45‐0.64)	<0.001
Liver resection	2974 (16.1)	0.30 (0.29‐0.32)	<0.001	0.37 (0.31‐0.45)	<0.001
Liver transplantation	1505 (8.1)	0.15 (0.14‐0.17)	<0.001	0.17 (0.13‐0.23)	<0.001
Other surgery	733 (4.0)	0.59 (0.54‐0.65)	<0.001	0.86 (0.62 ‐ 1.19)	0.36
Race					
American Indian/Alaska Native	201 (1.1)	1 (reference)	<0.001	1 (reference)	<0.001
White	12732 (68.7)	0.99 (0.83‐1.19)	0.93	0.88 (0.56‐1.39)	0.58
Black	2228 (12.0)	1.10 (0.91‐1.33)	0.31	0.97 (0.60‐1.56)	0.89
Asian or Pacific Islander	3360 (18.1)	0.70 (0.58‐0.84)	<0.001	0.48 (0.30‐0.77)	0.002
Marriage status					
Widowed	1931 (10.9)	1 (reference)	<0.001		
Divorced or separated or single	6026 (33.9)	0.76 (0.71‐0.81)	<0.001		
Married	9836 (55.3)	0.62 (0.59‐0.66)	<0.001		
Insurance status (Uninsured/Insured)	463 (3.8)/11628 (96.2)	0.64 (0.57‐0.73)	<0.001		

OS, overall survival; HR, hazard ratio; AFP, alpha‐fetoprotein; RFA, radiofrequency ablation.

To clearly distinguish the impact of HCC rather than comorbidities, the univariate and multivariate analyses for DSS in the general population were repeated (Table S1). The results were similar to those of the OS analyses. The median DSS (95% CI) of the ≤30 mm group, 31‐50 mm group, and >50 mm group were 86.0 (NA) months, 39.0 (35.8‐42.2) months, and 13.0 (12.0‐14.0) months, respectively. Additionally, tumor size was an outstanding and independent prognostic factor for DSS in the general population. Details are presented in Table S1 and the survival curves for DSS stratified by tumor sizes are shown in Figure S1.

### 3.3 Prognostic ability of tumor sizes in subgroups stratified by vascular invasion and surgery type

In the 7th edition of the AJCC TNM staging system for HCC, solitary tumors without vascular invasion were classified as T1, while those with vascular invasion were classified as T2. It was interesting to determine whether tumor sizes could contribute to a further refined T classification. Moreover, the choice of the three main surgery types had certain interactions with tumor sizes on HCC patients’ survival. Therefore, the prognostic ability of tumor sizes on OS was further explored in subgroups stratified by vascular invasion (No/Yes) and surgery type (RFA/LR/LT). The results of the multivariate analyses are listed in Table 3.

**Table 3:** Multivariate Cox proportional hazards regression analyses for the impact of tumor size on OS stratified by vascular invasion and surgery type

Group	Tumor size (mm)	Without Vascular invasion			*P* value	With Vascular invasion			*P* value
		No. (%)	No. of events (%)	Adjusted HR and 95% CI		No. (%)	No. of events (%)	Adjusted HR and 95% CI	
General	≤ 30	6497 (39.3)	2846 (43.8)	1 (reference)	<0.001	622 (30.4)	276 (44.4)	1 (reference)	<0.001
	31‐50	4497 (27.2)	2580 (57.4)	1.34 (1.27‐1.41)	<0.001	509 (24.9)	308 (60.5)	1.67 (1.41‐1.97)	<0.001
	> 50	5554 (33.6)	4089 (73.6)	2.01 (1.91‐2.12)	<0.001	912 (44.6)	632 (69.3)	2.19 (1.87‐2.56)	<0.001
RFA	≤ 30	1389 (61.8)	531 (38.2)	1 (reference)	<0.001	81 (61.8)	36 (44.4)	1 (reference)	0.18
	31‐50	663 (29.5)	376 (56.7)	1.43 (1.25‐1.64)	<0.001	30 (22.9)	22 (73.3)	1.70 (0.97‐2.98)	0.07
	> 50	194 (8.6)	123 (63.4)	1.62 (1.33‐1.98)	<0.001	20 (15.3)	15 (75.0)	1.37 (0.70‐2.67)	0.35
LR	≤ 30	706 (30.6)	235 (33.3)	1 (reference)	0.38	108 (16.2)	43 (39.8)	1 (reference)	0.06
	31‐50	666 (28.9)	230 (34.5)	1.05 (0.88‐1.26)	0.59	164 (24.6)	75 (45.7)	1.39 (0.96‐2.03)	0.08
	> 50	934 (40.5)	382 (40.9)	1.12 (0.95‐1.32)	0.17	396 (59.3)	202 (51.0)	1.49 (1.07‐2.08)	0.02
LT	≤ 30	906 (68.7)	687 (24.2)	1 (reference)	0.57	118 (63.4)	30 (25.4)	1 (reference)	0.01
	31‐50	351 (26.6)	78 (22.2)	0.87 (0.67‐1.13)	0.29	56 (30.1)	24 (42.9)	1.86 (1.08‐3.21)	0.03
	> 50	62 (4.7)	17 (27.4)	0.97 (0.59‐1.59)	0.90	12 (6.5)	13 (76.5)	3.75 (1.41‐10.00)	0.01

OS, overall survival; HR, hazard ratio; CI, confidence interval; RFA, radiofrequency ablation; LR, liver resection; LT, liver transplantation.

Multivariate analysis was adjusted by age (≤63/>63), year of diagnosis (2004‐2007/2008‐2010/2011‐2013), N classification (AJCC 6th, N0/N1), M classification (AJCC 6th, M0/M1), and surgery type (No surgery/Radiofrequency ablation/Liver resection/Liver transplantation/Other surgery) for general patients stratified by vascular invasion. For patients stratified by vascular invasion and surgery type, multivariate analysis was adjusted by age (≤63/>63), year of diagnosis (2004‐2007/2008‐2010/2011‐2013), N classification (AJCC 6th, N0/N1), and M classification (AJCC 6th, M0/M1). Alpha‐fetoprotein level, fibrosis score, race, marriage status, and insurance status were not included in multivariate analysis, because there was a considerable portion of patients with missing data in these characteristics and the sample size for positive vascular invasion subgroups was too small.

**Figure 3:**
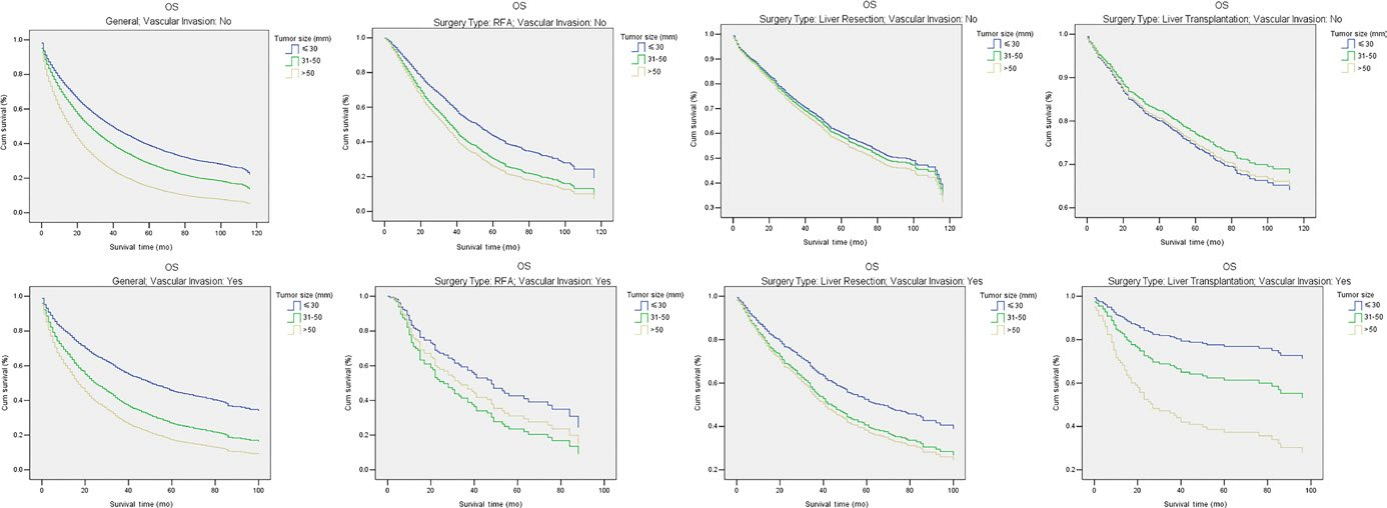
Subgroup analyses for the impact of tumor size (≤30 mm/31‐50 mm/>50 mm) on OS stratified by vascular invasion (No/Yes) and surgery type (RFA/LR/LT). Comparisons of OS for patients with different tumor sizes were conducted in subgroups stratified by vascular invasion (No/Yes) and surgery type (RFA/LR/LT). AHR and 95% CI are shown in Table 3. OS, overall survival; RFA, radiofrequency ablation; LR, liver resection; LT, liver transplantation; AHR, adjusted hazard ratio; CI, confidence interval

Tumor size remained an independent and striking prognostic factor for OS regardless of the status of vascular invasion (No/Yes) (all *P* < 0.001). In RFA patients without vascular invasion, tumor sizes were independent and distinct prognostic indicators for OS (all *P* < 0.001), while in those with vascular invasion, a trend of worse OS was only found in the 31‐50 mm group compared with the ≤30 mm group (HR and 95%CI: 1.70 [0.97‐2.98], *P* = 0.07). In contrast, for LR patients without vascular invasion, tumor sizes had no prognostic impact on OS, but for those with vascular invasion, the 31‐50 mm group had a trend of worse OS (*HR* and 95%CI: 1.39 [0.96‐2.03], *P* = 0.08) compared to the ≤30 mm group, and the >50 mm group had obviously worse OS (HR and 95%CI: 1.49 [1.07‐2.08], *P* = 0.02). Furthermore, the prognostic ability of tumor sizes in LT patients was similar to that of LR patients. For LT patients without vascular invasion, tumor sizes had no prognostic impact on OS. In LT patients with vascular invasion, compared with the ≤30 mm group, the other two groups had observably worse OS (HR and 95%CI: 1.86 [1.08‐3.21] and 3.75 [1.41‐10.00], *P* = 0.03 and *P* = 0.01, respectively). Detailed results of multivariate analyses for OS in various subgroups are presented in Table 3. The adjusted survival curves of OS stratified by vascular invasion and surgery type are shown in Figure 3.

The sample size was reduced in the analyses for DSS (N = 16 032) compared with those for OS (N = 18 591). Due to the further limited number of patients in the vascular invasion subgroup, only the prognostic impact of tumor sizes on DSS was analyzed in subgroups stratified by vascular invasion (no/yes), rather than surgery type. It was found that, regardless of whether there was vascular invasion, the other two groups had notably worse DSS compared with ≤30 mm groups (All *P* < 0.001). The adjusted survival curves of DSS stratified by vascular invasion are shown in Figure S2.

## 4 DISCUSSION

The current study explored the clinical significance of tumor sizes in solitary HCC classified as T1 and T2 by the 7th AJCC TNM staging system. It demonstrated that tumor size was a prominent and independent prognostic factor for HCC with or without vascular invasion in the whole population, which was associated with unfavorable clinicopathologic characteristics in solitary HCC. However, the prognostic ability of tumor sizes in patients receiving different surgery types (RFA/LR/LT) was dependent on the status of vascular invasion.

In RFA patients without vascular invasion, both tumors between 31‐50 mm and tumors >50 mm were associated with obviously inferior OS compared to tumors ≤30 mm. By contrast, in RFA patients with vascular invasion, a trend of worse OS was identified only in the 31‐50 mm group compared with the ≤30 mm group. Though it is not statistically significant, the adjusted survival curves of three subgroups in RFA patients with vascular invasion were separated as shown in Figure 3. It most likely resulted from the small sample size of subgroups (N = 36 in ≤30 mm group, N = 22 in 31‐50 mm group, and N = 15 in >50 mm group).

For nonvascular invasion patients receiving LR or LT, tumor sizes were not prominent prognostic indicators. Whereas in LT patients with vascular invasion, tumor sizes showed independent and notable prognostic ability. Similarly, in LR patients with vascular invasion, compared with the ≤30 mm group, the 31‐50 mm group showed a trend of worse OS, and the >50 mm group had markedly worse OS. The small sample size of LR patients with vascular invasion (N = 43 in ≤30 mm group, N = 75 in 31‐50 mm group, and N = 202 in >50 mm group) might have led to the lack of statistical significance in comparing the ≤30 mm group and the 31‐50 mm group.

Herein, it is concluded that tumor size was an important prognostic factor for HCC patients receiving RFA. However, for patients receiving LR or LT, tumor size was only a prognostic element for those with vascular invasion. These findings were consistent with some current consensus in clinical practice and existing research. According to related literature, the minimum ablative margin of RFA was proposed to be 10 mm. On the basis of the standard killzone as 50 mm, in order to achieve eradication theoretically, the maximum tumor size in RFA patients should be 30 mm, which has been demonstrated by many clinical reports.^24^, ^25^, ^26^ Although the optimal cut‐off value in tumor size for RFA is still controversial,^24^ it is convincing that tumor size has a remarkable influence on the outcome of RFA, which was supported by this study as well. Furthermore, current criteria for LT, such as the Milan criteria and United Network for Organ Sharing (UNOS) criteria, put emphasis on the absence of macrovascular invasion. In the current study, it was found that for LT patients without vascular invasion, tumor sizes had no appreciable effect on patients’ survival. However, for LT patients with vascular invasion, with increased tumor sizes, patients’ survival decreased dramatically, indicating that LT should be performed in these patients cautiously.

Taking into consideration the consistent prognostic ability of tumor sizes in vascular invasion patients receiving LR or LT, it was suggested that solitary HCC of the T2 classification could be further subdivided by tumor sizes to predict outcomes more accurately. Several previous studies regarding LR supported this proposal. Goh BK et al reported that in solitary HCC patients receiving LR, tumor size was an important predictive indicator for survival in the T2 classification but not in T1.^27^ Zhang et al demonstrated that for solitary HCC patients without vascular invasion, tumor sizes did not affect tumor recurrence and survival when they received LR.^28^ Compared with these studies, the current study had a relatively large sample size. Besides, RFA and LT patients were also included in this investigation, meaning it was not restricted to just LR patients.

For nonvascular invasion patients, tumor sizes had no impact on their survival regardless of receiving LR or LT. Therefore, in such patients, tumor size should not act as a reference factor in selection between LR and LT. According to the National Comprehensive Cancer Network (NCCN) guideline for HCC, the optimal tumor characteristic for LR is solitary tumor without major vascular invasion, rather than a specified limitation on tumor sizes. However, only those meeting UNOS criteria (single tumor ≤50 mm or 2‐3 tumors ≤30 mm, no macrovascular involvement, no extrahepatic metastasis) are recommended for LT. Although some studies had suggested conducting LT for larger HCC, their conclusions were debatable.^29^, ^30^, ^31^ This study disclosed that in solitary HCC patients without vascular invasion, tumors between 31‐50 mm and even tumors >50 mm had comparable prognoses with tumors ≤30 mm. Thus, for such patients, tumor size should not be a limiting factor for LT. Preoperative examinations such as computed tomography (CT) scans or magnetic resonance imaging (MRI) could only identify macrovascular involvement, while microvascular invasion is conventionally confirmed by postoperative pathological examination. Nowadays, though technically immature, several advanced methods have been developed to predict vascular invasion in HCC patients.^32^, ^33^ The further maturity of relevant technologies could possibly bring breakthroughs to clinical practice.

Vascular invasion has been considered an important prognostic indicator in HCC.^6^, ^7^, ^8^, ^9^ However, based on this study, no prognostic ability for vascular invasion was found in the whole population. Certain confounding factors were considered, such as surgery type diminishing vascular invasion's prognostic ability in the general population. To verify this assumption, the prognostic ability in each subgroup divided by surgery type was investigated. It was found that vascular invasion was associated with inferior OS in patients receiving LR (*P* < 0.001) and LT (*P* = 0.06). Hence, the data were fundamentally consistent with previous reports regarding the prognostic ability of vascular invasion.

As previously mentioned, the major limitation of the current study was its small sample size in positive vascular invasion subgroups stratified by surgery type. For instance, the sample size of RFA patients with vascular invasion was too limited to achieve statistical significance. In addition, this study was based on SEER registration, and data heterogeneity was more extensive than those from single centers. At the same time, some important prognostic factors, including liver function, comorbidities, and etiology factors were missing in the SEER database. As a result, the prognostic ability of tumor sizes could not be adjusted by these factors in the current study. Finally, the findings were not validated in another independent cohort, especially for patients receiving liver transplantation. A well‐designed multicenter study with greater patient enrollment should be performed to further confirm these results. Nevertheless, this study demonstrated the prognostic ability of tumor sizes in solitary HCC was of great significance, dependent on the status of vascular invasion and surgery type.

## 5 CONCLUSION

In the current study, tumor size was found to be associated with unfavorable clinicopathologic characteristics, and it was a significant prognostic indicator in solitary HCC. However, its prognostic ability was dependent on the status of vascular invasion and surgery type. For LR or LT patients, tumor size was not an independent prognostic factor for those without vascular invasion, but it had notable prognostic ability for those with vascular invasion. As suggested by this study, tumor size should be integrated into T2 classification for solitary HCC, and tumor size should not be a consideration in the choice between LR and LT for solitary HCC patients without vascular invasion.

## ETHICAL STATEMENT

This study was deemed exempt from institutional review board approval by The First Affiliated Hospital of Sun Yat‐sen University, thus informed consent was waived. The study was conducted in accordance with the ethical standards of World Medical Association Declaration of Helsinki.

## CONFLICTS OF INTEREST

None Declared.

## References

[1] McGlynn KA , Petrick JL , London WT . Global epidemiology of hepatocellular carcinoma: an emphasis on demographic and regional variability. Clin Liver Dis. 2015;19:223‐238. 25921660 10.1016/j.cld.2015.01.001 PMC4712629

[2] El‐Serag HB , Kanwal F . Epidemiology of hepatocellular carcinoma in the United States: where are we? Where do we go? Hepatology. 2014;60:1767‐1775. 24839253 10.1002/hep.27222 PMC4211957

[3] Maluccio M , Covey A . Recent progress in understanding, diagnosing, and treating hepatocellular carcinoma. CA Cancer J Clin. 2012;62:394‐399. 23070690 10.3322/caac.21161

[4] Zhang BH , Yang BH , Tang ZY . Randomized controlled trial of screening for hepatocellular carcinoma. J Cancer Res Clin Oncol. 2004;130:417‐422. 15042359 10.1007/s00432-004-0552-0 PMC12161851

[5] Zhang W , Wang X , Jiang R , et al. Effect of tumor size on cancer‐specific survival in small hepatocellular carcinoma. Mayo Clin Proc. 2015;90:1187‐1195. 26231292 10.1016/j.mayocp.2015.06.018

[6] Krasnodebski M , Grat M , Masior L , Patkowski W , Krawczyk M . Differential impact of risk factors for tumor recurrence in hepatitis B and hepatitis C virus‐infected patients undergoing liver transplantation for hepatocellular carcinoma. Ann Transplant. 2015;20:70‐75. 25639825 10.12659/AOT.892395

[7] Mokdad AA , Singal AG , Marrero JA , Zhu H , Yopp AC . Vascular invasion and metastasis is predictive of outcome in barcelona clinic liver cancer stage C hepatocellular carcinoma. J Natl Compr Canc Netw. 2017;15:197‐204. 28188189 10.6004/jnccn.2017.0020

[8] Jonas S , Bechstein WO , Steinmuller T , et al. Vascular invasion and histopathologic grading determine outcome after liver transplantation for hepatocellular carcinoma in cirrhosis. Hepatology. 2001;33:1080‐1086. 11343235 10.1053/jhep.2001.23561

[9] Hsieh CH , Wei CK , Yin WY , et al. Vascular invasion affects survival in early hepatocellular carcinoma. Mol Clin Oncol. 2015;3:252‐256. 25469305 10.3892/mco.2014.420 PMC4251122

[10] Berzigotti A , Reig M , Abraldes JG , Bosch J , Bruix J . Portal hypertension and the outcome of surgery for hepatocellular carcinoma in compensated cirrhosis: a systematic review and meta‐analysis. Hepatology. 2015;61:526‐536. 25212123 10.1002/hep.27431

[11] Jang CW , Kwon HJ , Kong H , et al. Impact of clinically significant portal hypertension on surgical outcomes for hepatocellular carcinoma in patients with compensated liver cirrhosis: a propensity score matching analysis. Ann Hepatobiliary Pancreat Surg. 2016;20:159‐166. 28261694 10.14701/ahbps.2016.20.4.159 PMC5325151

[12] Xiaohong S , Huikai L , Feng W , Ti Z , Yunlong C , Qiang L . Clinical significance of lymph node metastasis in patients undergoing partial hepatectomy for hepatocellular carcinoma. World J Surg. 2010;34:1028‐1033. 20174806 10.1007/s00268-010-0400-0

[13] Ikegami T , Yoshizumi T , Kawasaki J , et al. Surgical resection for lymph node metastasis after liver transplantation for hepatocellular carcinoma. Anticancer Res. 2017;37:891‐895. 28179348 10.21873/anticanres.11395

[14] Hasegawa K , Makuuchi M , Kokudo N , et al. Impact of histologically confirmed lymph node metastases on patient survival after surgical resection for hepatocellular carcinoma: report of a Japanese nationwide survey. Ann Surg. 2014;259:166‐170. 23532111 10.1097/SLA.0b013e31828d4960

[15] Kao WY , Su CW , Chiou YY , et al. Hepatocellular carcinoma: nomograms based on the albumin‐bilirubin grade to assess the outcomes of radiofrequency ablation. Radiology. 2017;285:670‐680. 28562211 10.1148/radiol.2017162382

[16] Gui B , Weiner AA , Nosher J , et al. Assessment of the Albumin‐Bilirubin (ALBI) grade as a prognostic indicator for hepatocellular carcinoma patients treated with radioembolization. Am J Clin Oncol. 2018;41:861–866. 28418940 10.1097/COC.0000000000000384 PMC5645222

[17] Deng Y , Pang Q , Miao RC , et al. Prognostic significance of pretreatment albumin/globulin ratio in patients with hepatocellular carcinoma. Onco Targets Ther. 2016;9:5317‐5328. 27601923 10.2147/OTT.S109736 PMC5005008

[18] Minagawa M , Ikai I , Matsuyama Y , Yamaoka Y , Makuuchi M . Staging of hepatocellular carcinoma: assessment of the Japanese TNM and AJCC/UICC TNM systems in a cohort of 13,772 patients in Japan. Ann Surg. 2007;245:909‐922. 17522517 10.1097/01.sla.0000254368.65878.da PMC1876960

[19] Chan AC , Fan ST , Poon RT , et al. Evaluation of the seventh edition of the American Joint Committee on Cancer tumour‐node‐metastasis (TNM) staging system for patients undergoing curative resection of hepatocellular carcinoma: implications for the development of a refined staging system. HPB. 2013;15(6):439‐448. 23659567 10.1111/j.1477-2574.2012.00617.x PMC3664048

[20] Vauthey JN , Lauwers GY , Esnaola NF , et al. Simplified staging for hepatocellular carcinoma. J Clin Oncol. 2002;20:1527‐1536. 11896101 10.1200/JCO.2002.20.6.1527

[21] Bradburn MJ , Clark TG , Love SB , Altman DG . Survival analysis part II: multivariate data analysis–an introduction to concepts and methods. Br J Cancer. 2003;89:431‐436. 12888808 10.1038/sj.bjc.6601119 PMC2394368

[22] Chen MS , Li JQ , Zheng Y , et al. A prospective randomized trial comparing percutaneous local ablative therapy and partial hepatectomy for small hepatocellular carcinoma. Ann Surg. 2006;243:321‐328. 16495695 10.1097/01.sla.0000201480.65519.b8 PMC1448947

[23] Giannini EG , Marenco S , Borgonovo G , et al. Alpha‐fetoprotein has no prognostic role in small hepatocellular carcinoma identified during surveillance in compensated cirrhosis. Hepatology. 2012;56:1371‐1379. 22535689 10.1002/hep.25814

[24] Kutlu OC , Chan JA , Aloia TA , et al. Comparative effectiveness of first‐line radiofrequency ablation versus surgical resection and transplantation for patients with early hepatocellular carcinoma. Cancer‐Am Cancer Soc. 2017;123:1817‐1827. 10.1002/cncr.30531 28085184

[25] Gory I , Fink M , Bell S , et al. Radiofrequency ablation versus resection for the treatment of early stage hepatocellular carcinoma: a multicenter Australian study. Scand J Gastroenterol. 2015;50:567‐576. 25615260 10.3109/00365521.2014.953572

[26] Weis S , Franke A , Mossner J , Jakobsen JC , Schoppmeyer K . Radiofrequency (thermal) ablation versus no intervention or other interventions for hepatocellular carcinoma. Cochrane Database Syst Rev. 2013;19:CD3046. 10.1002/14651858.CD003046.pub3 PMC11931681 24357457

[27] Goh BK , Teo JY , Chan CY , et al. Importance of tumor size as a prognostic factor after partial liver resection for solitary hepatocellular carcinoma: Implications on the current AJCC staging system. J Surg Oncol. 2016;113:89‐93. 26611492 10.1002/jso.24099

[28] Zhang H , Yuan SX , Dai SY , et al. Tumor size does not independently affect long‐term survival after curative resection of solitary hepatocellular carcinoma without macroscopic vascular invasion. World J Surg. 2014;38:947‐957. 24258262 10.1007/s00268-013-2365-2

[29] Mazzaferro V , Chun YS , Poon RT , et al. Liver transplantation for hepatocellular carcinoma. Ann Surg Oncol. 2008;15:1001‐1007. 18236119 10.1245/s10434-007-9559-5 PMC2266786

[30] Duffy JP , Vardanian A , Benjamin E , et al. Liver transplantation criteria for hepatocellular carcinoma should be expanded: a 22‐year experience with 467 patients at UCLA. Ann Surg. 2007;246:502‐511. 17717454 10.1097/SLA.0b013e318148c704 PMC1959350

[31] Zaydfudim VM , Chapman WC , Nagorney DM . Challenges in patient selection for liver resection or transplantation in patients with hepatocellular carcinoma beyond Milan criteria. Hepatobiliary Surg Nutr. 2017;6:287‐289. 28848757 10.21037/hbsn.2017.07.04 PMC5554769

[32] Lin CY , Liao CW , Chu LY , et al. Predictive value of 18F‐FDG PET/CT for vascular invasion in patients with hepatocellular carcinoma before liver transplantation. Clin Nucl Med. 2017;42:e183–e187. 28114226 10.1097/RLU.0000000000001545

[33] Baheti AD , Dunham GM , Ingraham CR , et al. Clinical implications for imaging of vascular invasion in hepatocellular carcinoma. Abdom Radiol. 2016;41:1800‐1810. 10.1007/s00261-016-0763-2 27142384

