# Supplementary material for: The power of tumor sizes in predicting the survival of solitary hepatocellular carcinoma patients
Source: Cancer Med. 2018 Nov 14;7(12):6040–50. doi: 10.1002/cam4.1873 (PMC6308097; doi:10.1002/cam4.1873)
Supplement: Supplementary file 1 [file CAM4-7-6040-s001.pdf]

Supplementary Table 1. Univariate and multivariate Cox proportional hazards regression analyses for the impact of tumor size on DSS.

Characteristics	Number (%)	Univariate		Multivariate	
		HR (95%*CI*)	*P* value	*Adjusted HR* (95%*CI*)	*P* value
Gender (Male/Female)	11890 (74.2) / 4142 (25.8)	1.01 (0.95 – 1.06)	0.83		
Age (≤ 63 / > 63)	9133 (57.0) / 6899 (43.0)	1.54 (1.47 – 1.62)	< 0.001	1.17 (1.02 – 1.35)	0.03
Year of diagnosis			< 0.001		0.002
2004 – 2007	4729 (29.5)	1 (reference)		1 (reference)	
2008 – 2010	5016 (31.3)	0.94 (0.89 – 0.99)	0.03	0.76 (0.62 – 0.94)	0.01
2011 – 2013	6287 (39.2)	0.84 (0.79 – 0.89)	< 0.001	0.68 (0.54 – 0.84)	< 0.001
Tumor size (mm)			< 0.001		< 0.001
≤ 30	6197 (38.7)	1 (reference)		1 (reference)	
31 – 50	4309 (26.9)	1.64(1.54 – 1.75)	< 0.001	1.77 (1.50 – 2.09)	< 0.001
> 50	5526 (34.5)	3.12 (2.95 – 3.31)	< 0.001	2.76 (2.32 – 3.28)	< 0.001
N classification (N0 / N1)	15490 (96.6) / 542 (3.4)	2.76 (2.50 – 3.06)	< 0.001	1.42 (1.00 – 2.00)	0.05
M classification (M0 / M1)	14997 (93.5) / 1035 (6.5)	4.27 (3.96 – 4.60)	< 0.001	3.32 (2.58 – 4.27)	< 0.001
Vascular invasion (No / Yes)	14242 (88.8) / 1790 (11.2)	1.01 (0.94 – 1.09)	0.74		
AFP level (Normal and Bordline/ Elevated)	4073 (25.4) / 8556 (78.8)	1.68 (1.58 – 1.78)	< 0.001	1.44 (1.24 – 1.67)	< 0.001
Fibrosis score (0 – 4 / 5 – 6)	1060 (22.4) / 3681 (77.6)	1.23 (1.09 – 1.39)	0.001	1.45 (1.19 -1.76)	< 0.001
Surgery type			< 0.001		< 0.001
No surgery	9406 (58.9)	1 (reference)		1 (reference)	
RFA	2019 (12.6)	0.37 (0.35 – 0.41)	< 0.001	0.54 (0.44 – 0.67)	< 0.001
Liver resection	2501 (15.7)	0.31 (0.28 – 0.33)	< 0.001	0.35 (0.28 – 0.44)	< 0.001
Liver transplantation	1407 (8.8)	0.09 (0.08 – 0.11)	< 0.001	0.08 (0.05 – 0.14)	< 0.001
Other surgery	637 (4.0)	0.55 (0.49 – 0.62)	< 0.001	0.64 (0.41 – 1.01)	0.05
Race			< 0.001		< 0.001
American Indian/Alaska Native	177 (1.1)	1 (reference)		1 (reference)	
White	10844 (67.9)	0.92 (0.74 – 1.15)	0.46	0.87 (0.50 – 1.51)	0.61
Black	1936 (12.1)	1.04 (0.83 – 1.30)	0.73	0.96 (0.54 – 1.71)	0.89
Asian or Pacific Islander	3009 (18.8)	0.68 (0.54 – 0.85)	0.001	0.49 (0.27 – 0.87)	0.01
Marriage status			< 0.001		
Widowed	1538 (10.0)	1 (reference)			
Divorced or separated or single	5395 (35.2)	0.73 (0.67 – 0.79)	< 0.001		
Married	8413 (54.8)	0.61 (0.57 – 0.66)	< 0.001		
Insurance status (Uninsured / Insured)	433 (4.2) / 9957 (95.8)	0.59 (0.52 – 0.68)	< 0.001		

Abbreviations: DSS, Disease-specific survival; HR, hazard ratio; AFP, alpha fetoprotein; RFA, radiofrequency ablation.

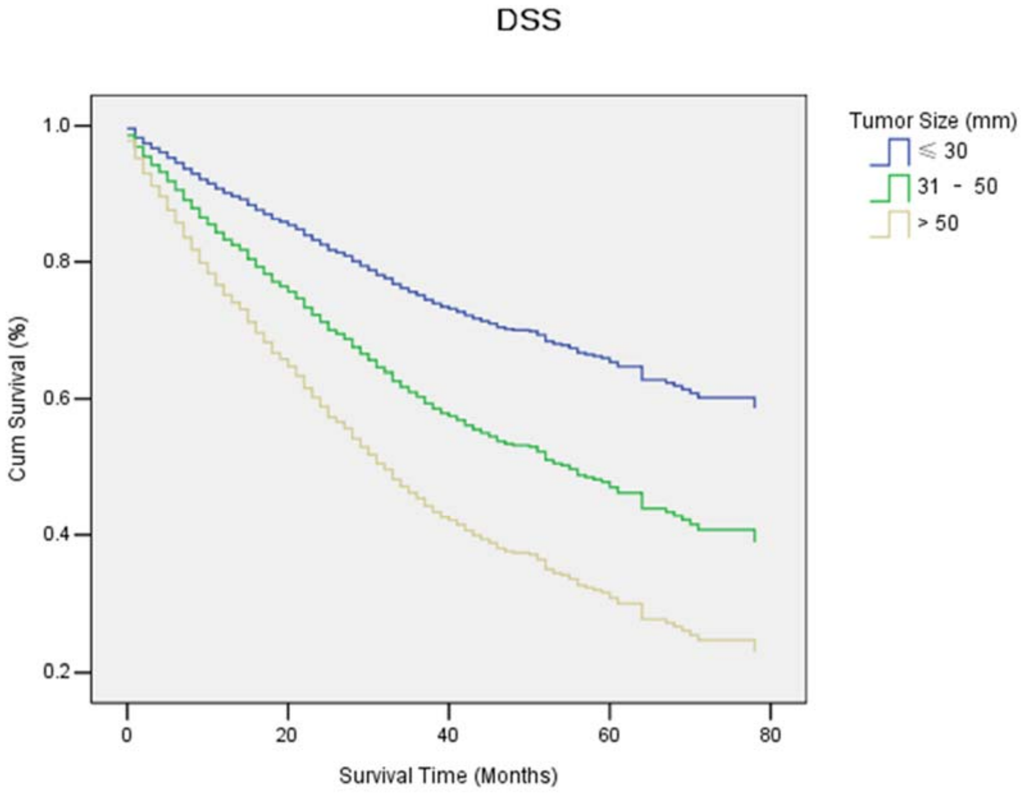

Supplementary Figure 1. Adjusted disease-specific survival by tumor size for the whole study population.

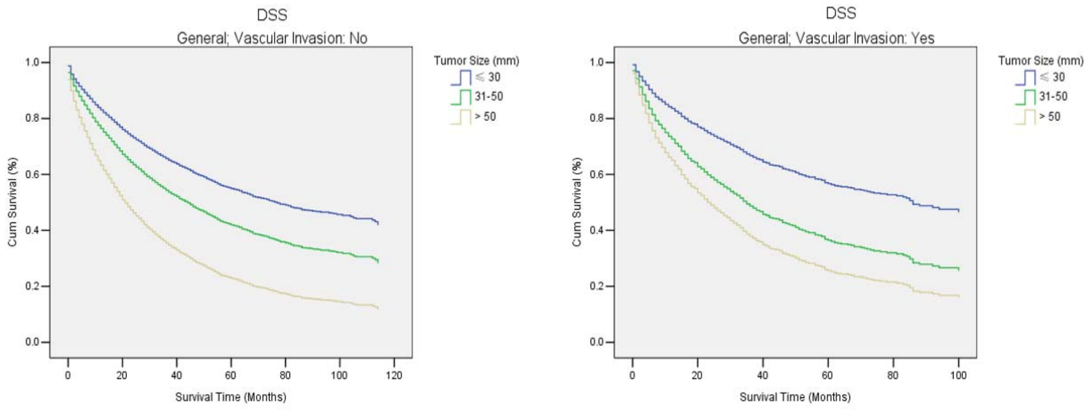

Supplementary Figure 2. Adjusted disease-specific survival by tumor size for patients with or without vascular invasion.
